# Monitoring a changing Arctic: Recent advancements in the study of sea ice microbial communities

**DOI:** 10.1007/s13280-021-01658-z

**Published:** 2021-11-25

**Authors:** Karley Campbell, Ilkka Matero, Christopher Bellas, Thomas Turpin-Jelfs, Philipp Anhaus, Martin Graeve, Francois Fripiat, Martyn Tranter, Jack Christopher Landy, Patricia Sanchez-Baracaldo, Eva Leu, Christian Katlein, C. J Mundy, Søren Rysgaard, Letizia Tedesco, Christian Haas, Marcel Nicolaus

**Affiliations:** 1 Department of Arctic and Marine Biology, UiT The Arctic University of Norway, Tromsø, Norway; 2 Bristol Glaciology Centre, School of Geographical Sciences, University of Bristol, Bristol, UK; 3 Alfred-Wegener-Institute Helmholtz Centre for Polar and Marine Research, Bremerhaven, Germany; 4 Department Geosciences, Environment and Society, Université Libre de Bruxelles, Brussels, Belgium; 5 Arctic Research Centre, Department of Bioscience, University of Aarhus, Aarhus, Denmark; 6 Department of Physics and Technology, UiT The Arctic University of Norway, Tromsø, Norway; 7 Akvaplan-Niva AS, CIENS, Gaustadalleen 21, 0349 Oslo, Norway; 8 Centre for Earth Observation Science, University of Manitoba, Winnipeg, MB Canada; 9 Greenland Climate Research Centre, Nuuk, Greenland; 10 Finnish Environment Institute, Helsinki, Finland

**Keywords:** Algae, Biogeochemistry, Climate Change, Microbes, Modeling, Sea ice

## Abstract

Sea ice continues to decline across many regions of the Arctic, with remaining ice becoming increasingly younger and more dynamic. These changes alter the habitats of microbial life that live within the sea ice, which support healthy functioning of the marine ecosystem and provision of resources for human-consumption, in addition to influencing biogeochemical cycles (e.g. air–sea CO_2_ exchange). With the susceptibility of sea ice ecosystems to climate change, there is a pressing need to fill knowledge gaps surrounding sea ice habitats and their microbial communities. Of fundamental importance to this goal is the development of new methodologies that permit effective study of them. Based on outcomes from the DiatomARCTIC project, this paper integrates existing knowledge with case studies to provide insight on how to best document sea ice microbial communities, which contributes to the sustainable use and protection of Arctic marine and coastal ecosystems in a time of environmental change.

**Supplementary Information:**

The online version contains supplementary material available at 10.1007/s13280-021-01658-z.

## Introduction

**Fig. 1:**
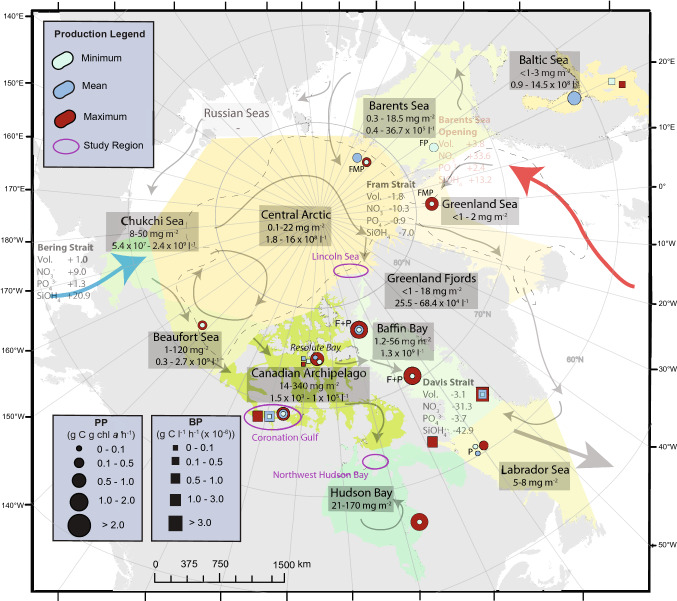
Regional summary of minimum (cyan), maximum (red) and average (dark blue) values for ^14^C-based algal primary production (PP, circles) relative to chlorophyll *a* (chl *a*) and bacterial production (BP, squares) in sea ice: first year (unlabeled or F), multiyear (M), pack ice of unspecified age (P), or a combination of types (e.g. F + P). Arrows indicate the main water inflows from the Pacific (blue) and Atlantic (red) Oceans, general movement of surface waters, and nutrient fluxes (kmol s^−1^) into (positive) and out of (negative) the Arctic. Ice algal chl *a* (boxes, mg m^−2^) and bacterial cell counts (boxes, cells l^−1^) are also specified. Arctic water bodies defined by the International Hydrographic Organization (1953) are shaded and regions of interest highlighted in this paper are circled (purple). The approximate boundary between continental shelf and deep-water basins is shown as a dashed line. See supplementary material (S.1) for further references of information

The Arctic marine system represents a diverse collection of water bodies that cover the continental shelves and deep-water basins of the northernmost latitudes on our planet (Fig. 1). A defining feature of these waters and the ecosystems they support is the presence of sea ice for at least some part of the year. While sea ice in the Arctic has shown rapid declines over recent decades (Meredith et al. 2019), the presence of liquid in sea ice throughout the year as saltwater brine inclusions, surface flooding, or meltwater ponds, continues to provide important habitat space for the growth of microorganisms like eukaryotic algae and prokaryotic bacteria (Mundy and Meiners 2021). These sympagic (i.e. ice-associated) communities plays a significant role in structuring the biogeochemical dynamics and food webs of the polar oceans, especially at a time when the ocean is still ice covered and phytoplankton growth in the ocean is limited by light (Lannuzel et al. 2020). Microbial adaptations for life in sea ice require modifications to intracellular processes, but also to extracellular controls. For example, this includes the exudation of gelatinous extracellular polymeric substances or production of ice-binding proteins that have been shown to modify the functioning of the microbial community and the structure of their ice environment (Krembs et al. 2011; Ewert and Deming 2013; Roukaerts et al. 2021). In this paper, we provide an overview on the complexities of sea ice microbial communities while introducing innovative methods that may be used to characterise their presence and function within Arctic sea ice. We also highlight the use of numerical models as tools to understand drivers of sea-ice algal phenology.

Understanding the critical role of sea ice microbial growth in Arctic marine ecology and biogeochemistry requires accurate knowledge on which microbial communities are present, how they function, as well as information on how abundant and active they are. However, analysing biological and biogeochemical properties in sea ice is fundamentally complicated by its inherent heterogeneity and multiphase nature (Miller et al. 2015), as well as the methodological limitations of studying this harsh environment. Traditionally, measurements of microbial composition have relied on the use of microscopy, which is suitable for the identification of eukaryotic organisms but can be biased by individual observers. Molecular biology techniques have the potential to allow rapid species identification in multiple environmental samples. The eukaryotic community can be identified by targeted sequencing of 18S ribosomal RNA (rRNA) genes. 18S rRNA genes are present in all eukaryotic organisms, but sequence variations between different groups exist, which allows the taxonomic composition to be determined. To target the prokaryotic community, 16S rRNA gene sequencing can be used to identify bacterial and archaeal members present in environmental samples in a similar manner (Caporaso et al. 2011). Further to this, random sequencing of all environmental DNA in a sample, using a technique called metagenomics, can allow the reconstruction of microbial genomes directly from environmental samples, allowing the gene content of specific prokaryotic organisms to be determined. It is through application of these molecular techniques that we may construct a more detailed library of all microorganisms and functions within sea ice.

As the dominant photosynthetic pigment of primary producers, chlorophyll *a* (chl *a*) is widely used to approximate ice algal abundance or even represent the primary productivity of a given location. Caution should be used interpreting the chl *a* proxy in isolation, as the concentration of this pigment relative to carbon can vary substantially with environmental conditions, algal acclimation state, and species (Falkowski and Raven 2007). It is for this reason we make the distinction of chl *a* biomass within this work, which is separate from biomass calculated as the dry weight of organic matter within a sample. The time-consuming and destructive collection of sea ice samples for chl *a* analysis prevents true time series measurements of ice algal blooms, and it is unlikely to capture the patchy distribution (i.e. spatial variability) inherent to sea ice algae (Campbell et al. 2015). Furthermore, procedures of ice melt during processing (Campbell et al. 2019) and sample preservation via freezing (Graff and Rynearson 2011) have been shown to artificially reduce the amount of chl *a* pigment subsequently measured by methods of fluorescence. Despite these disadvantages, the direct extraction of chl *a* from destructively sampled sea ice is a fundamental parameter in any assessment of sea ice biology, and as a result, methods to improve ease of chl *a* data collection via use of remote sensors (e.g. Mundy et al. 2007a, b) have improved characterisation of sea ice algal blooms. However, we are still lacking an observation-based estimate of sea ice primary production at the scale of Arctic sea ice. Accordingly, the contribution of the sympagic community to the primary production of the Arctic Ocean remains poorly understood. It is becoming a pressing need to better evaluate its contribution and variability in response to the rapidly changing environmental conditions.

Assessing the response of sea ice microbial communities to environmental change that is driven by amplified global warming in the Arctic (Meredith et al. 2019) requires the predictive capabilities of biogeochemical models. Such modeling has shown that the expected response of sea ice habitats to climate change in the Arctic is highly variable across regions and latitudes (Tedesco et al. 2019; Watanabe et al. 2019; Castellani et al. 2020). In addition to regional variability and local heterogeneity of ice algae communities, field observations of ice algae abundance and distribution are scarce (Miller et al. 2015). Furthermore, modelling approaches remain limited by the number of processes and functional groups that are included to represent a number of dynamic processes, which in turn control sea ice microbial production. One example is the representation of the algal community as a single functional group (e.g. diatoms) despite documented variability in composition (Gosselin et al. 1997; Campbell et al. 2015). Furthermore, the ability of models to determine ice algal bloom timing and magnitude requires accurate representation of complex growth conditions, such as nutrient supply as a function of both sub-ice water movement and diffusive processes (Duarte et al. 2021 and references therein) and light availability (Tedesco et al. 2019). The heterogeneity of bottom-ice light intensities poses a particular challenge for modelling of ice algal blooms, where integrating field observations can improve our parameterisation of light availability and provide new understanding on the sensitivity biogeochemical processes (e.g. chl *a* accumulation) to spatial variations in light availability.

Effective ecosystem-based management of Arctic waters must bring together current knowledge across trophic levels (i.e. from microorganisms to megafauna), while planning for data collection in the future that addresses critical knowledge gaps and the challenges of a rapidly changing system. Here we provide a summary of sympagic microbial communities of the Arctic, particularly on the abundance, function and production of prominently studied algal and bacterial groups of microbial life (Fig. 1). We highlight methodological developments that advance our study of these sea ice microbial communities based on outcomes of the Changing Arctic Ocean DiatomARCTIC (Autecological Responses with Changes To Ice Cover) project, which has worked to characterise the conditions of sea ice habitats and the resultant impacts of microbial communities within them from a species-specific (i.e. autecological) perspective. Methodological advances of the project include: (i) the application of molecular analyses for identification of community composition and function, (ii) the development of remote sensing techniques for quantification of ice algal chl *a* biomass, as well as (iii) the adaptation of the Biogeochemical Flux Model (BFM-SI) of Tedesco et al. (2010) for representation of ice algal bloom development as a function of physical–chemical growth conditions. We use data from contrasting regions of the Arctic to provide case studies of these methodological advancements (Fig. 1), with: molecular insights from northwestern Hudson Bay (2019); remote sensing highlights from Hudson Bay (2019), as well as the Lincoln Sea of the High Arctic (2018); and model-focused work from the Coronation Gulf of the Canadian Archipelago (2014). From this assessment we help characterise the dynamics of changing sea ice habitats within the Arctic marine ecosystem and highlight important method-related considerations in their future study.

## Sea ice microbial communities

Microbial life in sea ice is most active in spring, when algae rapidly colonise the bottommost centimeters of the ice following the return of sunlight (Leu et al. 2015). The abundance of sea ice algae varies strongly across the ice subsurface, and a number of studies have suggested a patchy distribution of chl *a* biomass on the order of 5 m (e.g. Rysgaard et al. 2001; Søgaard et al. 2010; Katlein et al. 2014). Additionally, there is significant variability in the chl *a* and productivity of sea ice algae between seasons and years of study (Leu et al. 2015), as well as across the different regions of the Arctic (Fig. 1; S1). The pan-Arctic variability in chl *a* and production, which is especially evident when comparing the magnitude of chl* a* and production between basins of the central Arctic (low chl *a* and production) and continental shelf regions like Baffin Bay (high chl *a* and production), is reflective of differences in ice dynamics and limitations on algal growth. For example, while Resolute Bay in the Canadian Arctic is distanced from nutrient-rich Pacific and Atlantic waters entering the comparatively nutrient-depleted surface waters of the Arctic Ocean, chl *a* and maximum algal production in the first-year ice present is still high due to local topography that mixes the water column and replenishes nutrients for algal growth (Michel et al. 2006).

The algal communities within sea ice demonstrate significant biodiversity, with over 1000 species having been reported to date in the Arctic (Poulin et al. 2011). Sea ice algae can be further divided based on the location of colonisation at the ice surface, within the subsurface (i.e. bottom-ice described above), internally, or loosely attached to the bottom-ice. Flagellates typically represent the most abundant algal functional group within surface and interior communities, while pennate diatoms like the common species *Nitzschia frigida* dominate the ice bottom (Leeuwe et al. 2018). The composition of sea ice algal communities is not static; a shifting dominance of species or functional groups in response to changing light, nutrient and salinity conditions has been documented within or between studies (Leeuwe et al. 2018). For example, centric diatoms have the potential to outcompete pennate forms under nutrient deplete-high light conditions more typical of late versus early spring (Campbell et al. 2018).

Similar to ice algae, the sea ice prokaryotic communities are abundant and diverse. They are mainly dependent on sea ice algal growth for their carbon requirements, and thus their abundance rapidly increases during the development of spring blooms when usable forms of organic carbon are released by growing algal cells (Arrigo 2014). Another peak of prokaryotic growth has been shown to be stimulated by the release of usable organic carbon with decay of sea ice algae during the termination phase of the spring bloom (Kaartokallio 2004). Sea ice bacteria are typically dominated by *Octadecabacter, Polaribacter* and *Glaciecola* genera. However, obligate anaerobic sulphate reducing bacteria like *Desulforhopalus* have been reported in Antarctic sea ice (Eronen-Rasimus et al. 2017), indicating a variety of metabolic strategies may exist. The full extent of metabolic strategies in sea ice is poorly understood, owing to relatively few molecular studies addressing the functional genes present in such prokaryotic communities.

Unicellular eukaryotic organisms are major consumers of microalgae and bacteria in sea ice. A wide variety have been documented, such as ciliates, flagellates and amoeboid forms. Through their grazing activities, they may release nutrients from the algal biomass and can serve as a food source for larger metazoans (Caron et al. 2017). Hence, their abundance is generally correlated with, or lags behind, changes in abundance of bacteria and algae in sea ice (Arrigo 2014.) In terms of multicellular grazing organisms, nematodes within sea ice may be especially abundant in regions like Resolute Bay, Canada, where ice algal blooms are often significant (Riemann and Sime-Ngando 1997; Fig. 1). Similarly, copepods grazing on the concentrated biomass of ice algal blooms can also be abundant (Michel et al., 2006).

## Algal and bacterial production in sea ice

Still with a lot of uncertainties, sea ice algal growth can represent up to 60% of the total primary production in ice-covered waters (Gosselin et al. 1997), with total productivity largely controlled by a combination of light and nutrient conditions that inherently vary across a range of spatio-temporal scales (Leu et al. 2015). For example, light transmitted through sea ice and thus to the bottom-ice algal layer is decaying exponentially with ice thickness and, more importantly, the depth of overlying snow cover (Perovich 1990). Nutrient availability is difficult to assess, as this depends both on how the brine channel system is connected with the underlying ocean, and how microscale processes like microbial recycling and the creation of microenvironments with distinct biogeochemical dynamics (e.g. Fripiat et al. 2017; Roukaerts et al. 2021) control nutrient concentrations in the brine. In addition, there are a number of other factors influencing nutrient availability, such as: regional proximity of the sea ice to Pacific or Atlantic inflow (Fig. 1; S1), conditions of water column stability (Duarte et al. 2021) that were described previously, as well as the density of algae within a given bloom (Campbell et al. 2014). Research has also increasingly demonstrated that algal speciation, as well as the salinity of surface and ice-ocean interface waters, are likely to influence bloom productivity (Campbell et al. 2018).

Differences in the biophysical conditions of sea ice habitats and the surface waters beneath them translates to variability in primary production of spring ice algal blooms across the Arctic (Fig. 1). Fewer measurements exist for sea ice prokaryotes like bacteria, and thus far less is understood about the controls of their growth. This includes an apparent lack of region-specific variability or clear relationship with primary production across the Arctic (Fig. 1), despite the understood importance of sea ice algae in supplying usable carbon. The net influence of algal and prokaryotic production on sea ice biogeochemical state is complex, where both net autotrophic (O_2_ production and CO_2_ consumption) and heterotrophic (O_2_ consumption and CO_2_ production) conditions have been documented during the spring bloom (Campbell et al. 2017; Campbell et al. in review). The potential for net heterotrophy and thus localised O_2_-limited conditions in sea ice could favor anaerobic metabolisms, such as facultatively anaerobic denitrification processes that may further reduce nitrogen availability to sea ice algae (Rysgaard et al. 2008). The influence of sea ice microbes on biogeochemical cycles remains poorly described for the polar regions as a result of gaps in data collection and the complexity of such algal–prokaryote interactions (Vancoppenolle and Tedesco 2017; Leeuwe et al. 2018).

## Molecular tools to assess community composition and metabolism (Northwest Hudson Bay, Canada)

### Eukaryotic diversity in sea ice via 18S ribosomal RNA gene sequencing

**Fig. 2:**
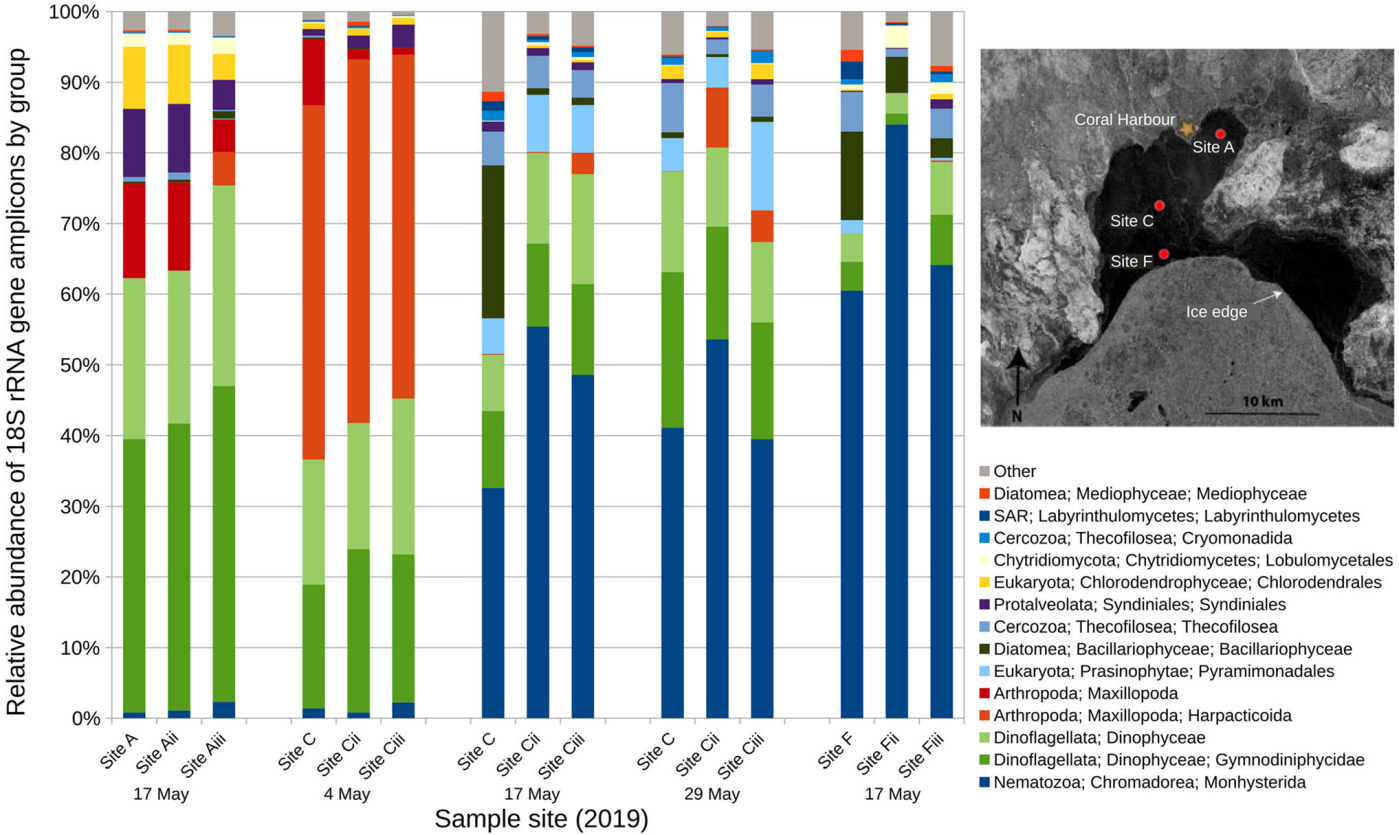
Relative abundance of 18S rRNA gene amplicons at three sea ice sites (A, C, F) in northwestern Hudson Bay, shown on synthetic aperture radar (SAR) image for 16 May, 2019, offshore from the community of Coral Harbour. Organisms > 10 mm were selected for in this analysis by filtration. Note that Site C was sampled at three time points (4th, 17th and 29th May). Three replicates were generated from each sampling location

A remarkably diverse community of eukaryotic organisms has been reported in sea ice through use of 18S rRNA gene amplicon sequencing (e.g. Bachy et al. 2011; Stecher et al. 2016). This molecular approach relies on detecting eukaryotic 18S rRNA genes of environmental samples, then assigning the segments of genetic material the closest identifiable species. We applied this technique to three sites (A, C, F) of bottom-ice sample collection in northwestern Hudson Bay (Fig. 1), in an effort to better document the presence of grazing microorganisms (> 10 µm) in data poor regions of the Arctic (see methods in S2). Through this assessment we found that dinoflagellates, which typically account for 4–16% of algal communities in sea ice (Poulin et al. 2011), were present in all samples. In particular, at sites A and C where they made up approximately 40–60% of 18S rRNA reads (Fig. 2) by relative abundance. The majority of dinoflagellates were identical to sequences (species) previously detected in High Arctic sea ice (Bachy et al. 2011). We also detected a large proportion of sequences (32–84%) that belonged to nematodes, particularly in 17 May samples, as well as a number of copepods (49–51%) on 4 May (Fig. 2). Both of these groups of grazing microorganisms contained genera with known salinity tolerance (Riemann and Sume-Ngando 1997), which is clearly advantageous for life in sea ice environments. In this case study of northwestern Hudson Bay, molecular methods proved a useful tool to assess the diversity of eukaryotic organisms. We have found that grazer community composition in the sea ice was highly variable between sites (A, B, C), as well as over the course of a single month (Fig. 2). After testing this methodology on sea ice ecosystems, 18S rRNA gene sequencing has shown the potential to perform high resolution assessments over short temporal and spatial scales in the future, providing an important first step in assessing grazer dynamics throughout ice algal blooms.

### Metabolic potential of the prokaryotic community

Similar to 18S rRNA gene amplicon sequencing of eukaryotes, identification of the prokaryotic community via 16S rRNA gene sequencing has yielded valuable insights into the composition of sea ice communities (e.g. Eronen-Rasimus et al. 2017). Knowledge on the metabolic function of these microbial communities can also be obtained if reference genomes are available (Langille et al. 2013), which for example, provides insight on the role of different organisms in biogeochemical cycling of elements. However, approaches like 16S rRNA gene sequencing are likely to be more difficult in sea ice environments because they may contain undocumented microbial strains that lack reference genomes. In addition, closely related bacterial strains with near-identical 16S rRNA gene amplicons (i.e. they group as one organism in this type of analysis) can have very different genomic DNA content, meaning we may be missing out on a full understanding of their encoded metabolic genes. This is exemplified by *Octadecabacter* genera occurring in both the Arctic and Antarctic sea ice which share > 99% identical 16S rRNA gene amplicons, yet only 42% of their genomic DNA (Vollmers et al. 2013). To uncover the metabolic pathways encoded by these potentially unique strains, metagenomics and the subsequent assembly of Metagenomic Assembled Genomes (MAGs) directly from environmental samples provides a means of more thoroughly documenting the sea ice prokaryotic community. In this way, individual prokaryotic genomes of bacteria or archaea can be fully or partially reconstructed, taxonomically identified, and have their functional genes annotated.

**Fig. 3:**
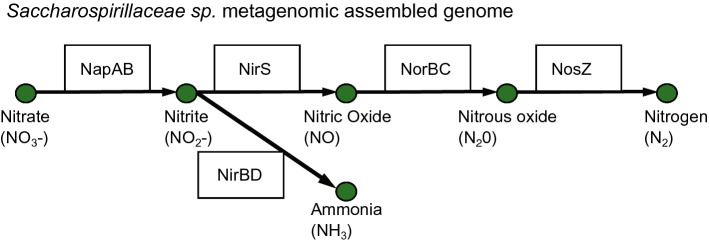
Example of denitrification pathway in a metagenomic assembled genome (MAG) of the Family Saccharospirillaceae. The MAG encodes both the facultatively anaerobic denitrification and dissimilatory nitrate reduction to ammonia pathways. It represented up to 10% of the prokaryotic community in northwestern Hudson Bay. Encoded enzymes in the MAG are designated in the black rectangles: *NapAB* nitrate reductase; *NirS* nitrite reductase; *NorBC* nitric oxide reductase subunits; *NosZ* nitrous-oxide reductase; *NirBD* nitrite reductase subunits

To test methodologies that uncover the metabolic potential of sea ice prokaryotic communities, metagenomics (the sequencing of genetic fragments from all organisms in an environmental sample) and the subsequent reconstruction of metagenomic assembled genomes (MAGs) was also performed on the samples of northwestern Hudson Bay (Fig. 1; Table S1). We found that one of the most abundant reconstructed bacterial genomes belonged to the Saccharospirillaceae family, and it encoded genes for both facultatively anaerobic denitrification as well as dissimilatory nitrate reduction to ammonia pathways (Fig. 3). The presence of such organisms supports previous reports of denitrification in sea ice (Rysgaard et al. 2008), suggesting that sea ice ecosystems in northwestern Hudson Bay may experience localised O_2_-limited conditions that can favour organisms capable of both aerobic and anaerobic respiration. Such conditions and metabolisms may increase the removal of nitrogen from sea ice and serve to enhance nutrient limitation within sea ice microbial communities. From this example, it is evident that metagenomics and the genomes identified are an important tool in investigating prokaryotic functional pathways and their role in the cycling of nutrients and carbon in sea ice habitats.

## Sensor-based assessment of sea ice algal chlorophyll *a* (Lincoln Sea and Northwest Hudson Bay, Canada)

Studies on sea ice microbial communities in the Arctic are limited by the logistics of accessing its more remote regions. For example, the concentrated number of primary production values within the vicinity of the Resolute Bay, Canada, research facility (*n* = 6) represents 27% of all measurements in Fig. 1, and only two other studies on sea ice primary production in the central Arctic Ocean (Wheeler et al. 1996; Gosselin et al. 1997) existed prior to the study of Boetius et al. (2013). Multi-national investments towards Arctic research over the last decade (e.g. 2019–2020 Multidisciplinary drifting Observatory for the Study of Arctic Climate (MOSAiC) drift campaign) continue to improve the representation of measurements across the Arctic. Combined with the time-consuming and destructive nature of processing sea ice samples, the development of remote sensing-based methods of measuring chl *a* have the potential to improve efficiency of sampling and ultimately the spatio-temporal coverage of sea ice biogeochemical studies.

**Fig. 4:**
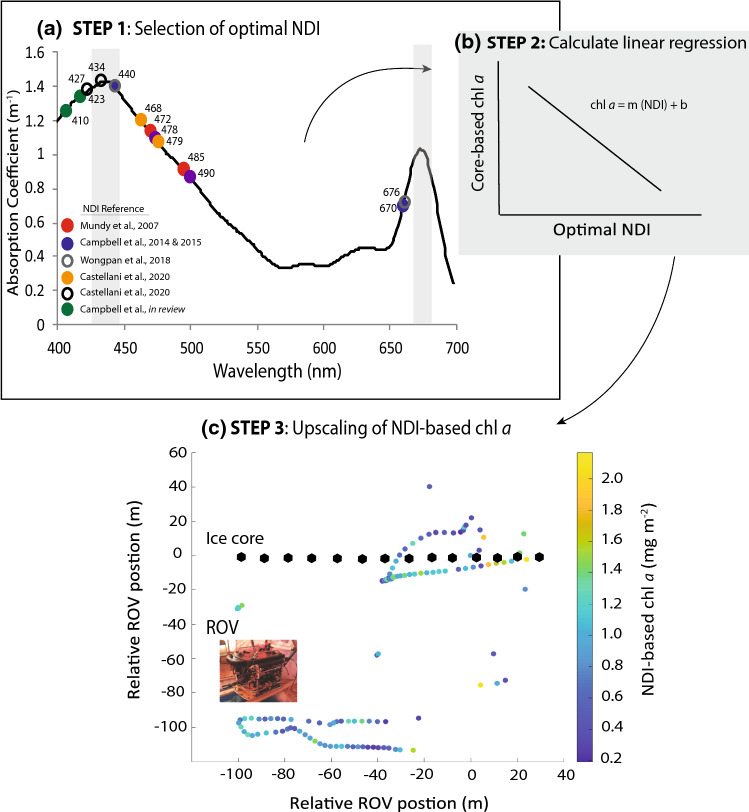
Illustration of process in using normalized difference indices (NDI) to remotely estimate ice algal chlorophyll *a* (chl *a*): **a** Step 1. An optimal NDI is calculated for a given study following Pearson Correlation analysis. The resultant optimal wavelength pairs of published NDIs are shown on the spectra of chl *a* absorption across photosynthetically active radiation (PAR), with 440 nm and 670 nm chl *a* absorption peaks highlighted (grey); **b** Step 2. A linear regression between ice core-based chl *a* and the optimal NDI is established for a given study; **c** Step 3. The linear regression of Step 2 may be applied to under-ice light data measured by remotely operated vehicle (pictured, ROV) to estimate chl *a* (coloured circles; secondary axis scale) over greater areas following calibration to ice cores (black circles). Data from Campbell et al. (*in review*)

An increasingly common approach to remotely estimate chl *a* across the sea ice subsurface is to position an upward looking hyperspectral sensor under the ice and assess the impact of pigment absorption on the spectra of transmitted photosynthetically active radiation (PAR). Chlorophyll *a* concentration can be related to a change in the shape of the transmitted PAR spectrum, as a result of the pigment’s preferential absorption of certain wavelengths (Fig. 4a). Since initial use of 671:540 nm ratio to describe this relationship (Legendre and Gosselin, 1991), a number of studies have improved the representativeness of this chl *a* proxy by calculating Normalised Difference Indices (NDIs) of particular wavelengths correlated to traditionally core-based chl *a* values. Here, NDIs are calculated according to the equation:

1 $${\text{NDI}}_{{\left( {\lambda 1, \, \lambda 2} \right)}} = \, {{\left( {T_{{z\left( {\lambda 1} \right)}} - \, T_{{z\left( {\lambda 2} \right)}} } \right)} \mathord{\left/ {\vphantom {{\left( {T_{{z\left( {\lambda 1} \right)}} - \, T_{{z\left( {\lambda 2} \right)}} } \right)} {\left( {T_{{z\left( {\lambda 1} \right)}} + \, T_{{z\left( {\lambda 2} \right)}} } \right)}}} \right. \kern-\nulldelimiterspace} {\left( {T_{{z\left( {\lambda 1} \right)}} + \, T_{{z\left( {\lambda 2} \right)}} } \right)}},$$

where, *T*_*z*(*λx*)_ represents transmitted irradiance or transmittance at a given wavelength of PAR (Mundy et al. 2007a). Due to variability in the concentration of accessory pigments and the presence of non-algal absorbing particles within sea ice, calibration and calculation of location-specific NDIs have been advised before their application in determining chl *a* from transmitted light alone (Campbell et al. 2015; Melbourne-Thomas et al. 2015). As a result, a number of different NDI wavelength combinations have been used to remotely assess sea ice chl *a* across the polar regions (Fig. 4a).

The value of this method is highlighted by recent work of the DiatomARCTIC project in the Lincoln Sea of the High Arctic (Fig. 1, 4c). Here, an optimal NDI of wavelengths 410 and 423 nm (NDI_(410, 423)_) was determined through Pearson Correlation analysis. A linear regression between ice core-based chl *a* and transmittance (Fig. 4b), at co-located sites (black points; Fig. 4c), was then determined. Finally, the linear regression from these steps was applied to remotely estimate chl* a* across the subsurface of the study ice floe (Fig. 4c; colours) using transmittance measured via a remotely operated vehicle (ROV; Katlein et al. 2017). Through this work, the spatial coverage of chl *a* data was broadened from for the individual core locations (Campbell et al. in review), and as a result, our understanding of spatial variability in the ice algal bloom was improved.

Assessment of spectral absorption is useful for estimating ice algal chl *a* for the entire ice column, which provides an integrated value for surface, interior and bottom-ice communities. However, the distribution of sea ice algae is also known to vary horizontally across fine (millimetres) spatial scales with the presence or absence of brine channel features (Mundy et al. 2007b), as well as vertically through the ice column (Gradinger 1999). This vertical positioning is of particular interest as it determines the susceptibility of ice algae to grazing and export processes at the ice bottom, and has implications for nutrient availability to the ice algae. At present, assessments on the vertical distribution of algae within sea ice habitats are limited to destructive sampling of chl *a*. However, new deployments of light sensors through the ice profile (e.g. Katlein et al. 2021) show promise for further development of non-destructive sensor-based estimates of sea ice algal chl *a*.

**Fig. 5:**
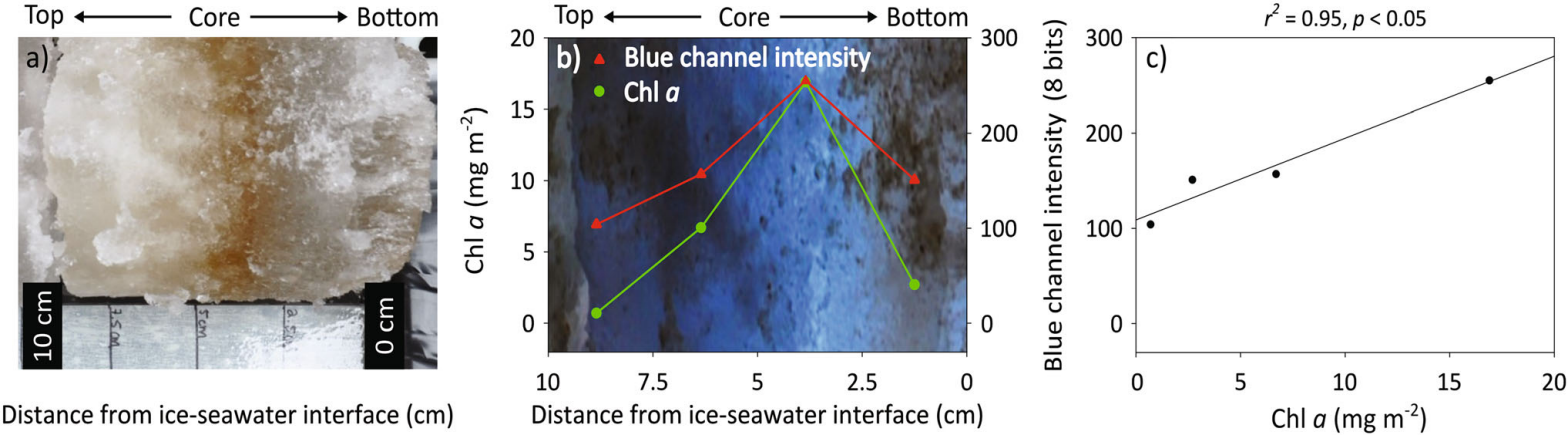
**a** A coloured band of algal pigments indicating the chlorophyll (chl) *a* biomass present in the bottom 10 cm of ice cores collected from northwestern Hudson Bay; **b** the chl *a* biomass, as well as 8-bit blue channel intensity is shown for 2.5-cm intervals; **c** the linear relationship between chl *a* biomass and blue channel intensity in the bottom 10 cm of sea ice

Many pennate forms of sea ice algae are able to move through the brine network by exuding adhesive extracellular polymeric substances. While the full extent of cell mobility remains largely unknown for sea ice algae (e.g. drivers of movement and speed), variability in the vertical distribution of cells through the ice that is observed as a change in location of the coloured band of pigment (Fig. 5), may be attributed in-part to such abilities. This movement can occur in response to changing light conditions, where the distance of ice algal bands from the ice-ocean interface has been found to be inversely related to bottom-ice light intensities (Aumack et al. 2014). Although space within the brine network, as well as strong gradients in temperature, salinity and nutrients away from the ice-ocean interface ultimately restrict the vertical extent of algal colonisation or movement in most instances. Similar to the association of NDIs to core-based chl *a*, we may also apply photometric approaches to quantify such movement. For example, using field observations of northwestern Hudson Bay (Fig. 1) we relate the vertical distribution of chl *a* biomass in the bottom-ice at 2.5-cm intervals to blue channel pixel intensity in 24-bit RGB images (Fig. 5a, b), which corresponds to wavelengths of strong chl *a* absorption (450–490 nm; Fig. 4a). From this work and the application of the resulting linear relationship between chl *a* and pixel intensity one may quantify fine scale changes in the vertical position of chl *a* from photographs alone (Fig. 5c).

## Biogeochemical modelling of sea ice algal production (Coronation Gulf, Canada)

### Modeling as a tool in research

Numerical models of biogeochemical processes have been developed to better study pan-arctic variability and potential future changes in sea ice primary production, nutrient and gas dynamics (Vancoppenolle and Tedesco 2017; Watanabe et al. 2019; Tedesco et al. 2019). One such model is the Biogeochemical Flux Model (BFM; Vichi et al. 2015), which represents the biogeochemistry of lower trophic levels in the marine environment. The sea ice extension of the BFM (i.e. BFM-SI; Tedesco et al. 2010) is one of the first biogeochemical 1D process models for sea ice that accounts for competition between algal groups, or for potential differences in light and nutrient acclimation states of the algae.

### Defining model function

**Fig. 6:**
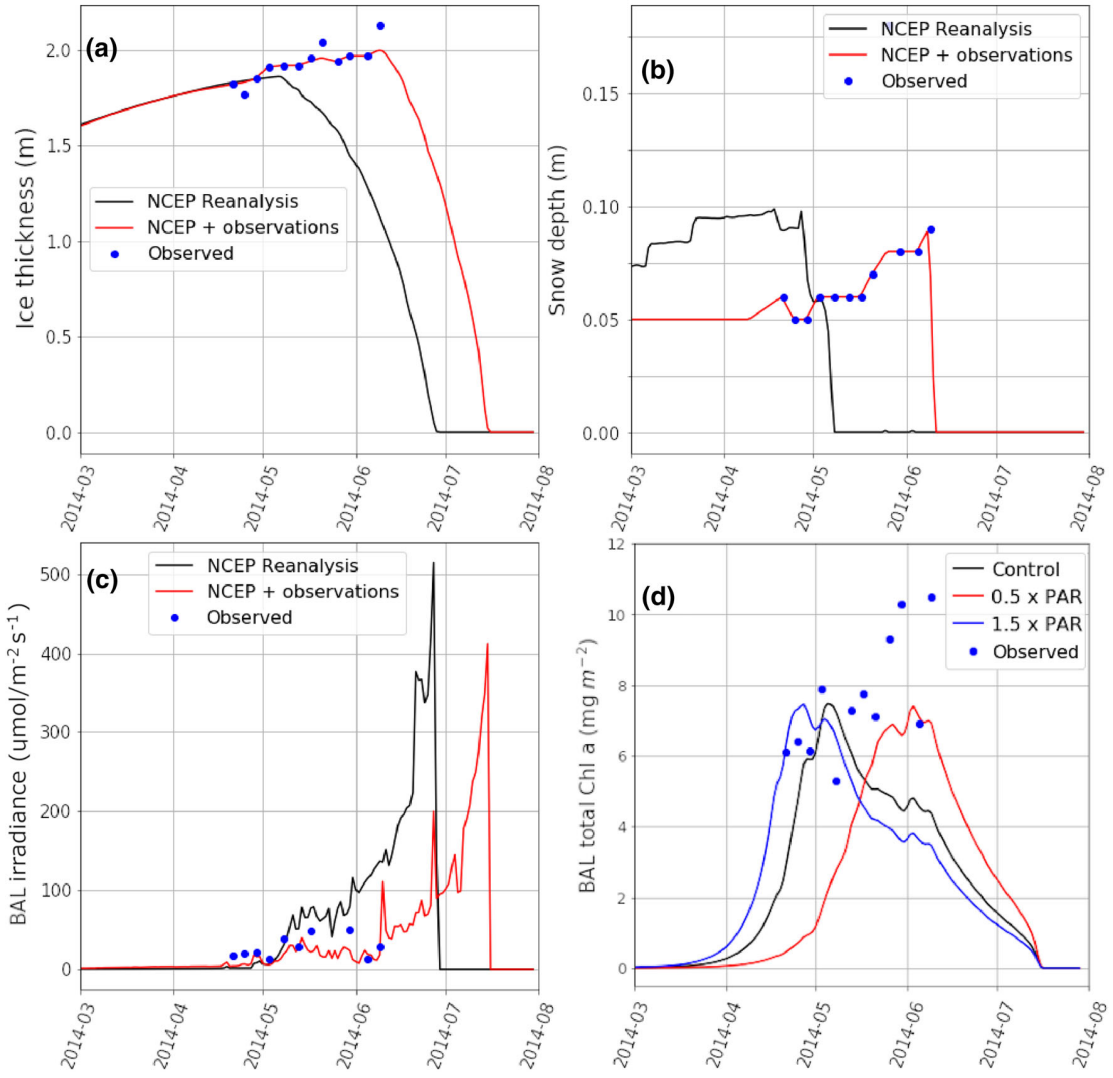
Model output of **a** sea ice thickness, **b** snow depth and **c** irradiance as quanta of light in the range of photosynthetically active radiation (PAR) in the Biologically Active Layer (BAL; Tedesco et al. 2010) for the study setup. Model output based on reanalysis data with and without using sea ice thickness and snow depth observations as input for the model shown in red and black respectively, with the field observations shown in blue. **d** The model sensitivity of total chl *a* in the biologically active layer to scaling the light levels up and down by 50%. The evolution of total chl *a* concentration in BAL under the modelled PAR irradiance levels is shown in black, and the results from the simulations in which the PAR levels are scaled down and up by 50% are shown in red and blue, respectively

This version of BFM-SI uses the NCEP/NCAR Reanalysis 1 data (Kalnay et al. 1996) as a forcing for the atmospheric variables. In-situ measurements of precipitation and surface air temperature can be used as an additional forcing to nudge the model towards a state observed on the field. As part of the DiatomARCTIC project we have further developed the model to include observations of snow depth and ice thickness, which in turn allows for more accurate determination of light transmission to the biologically active layer (BAL) of sea ice. In the model the BAL is defined as the continuous ice layer extending upwards from the ice–ocean interface, in which the brine volume is at or over 5% (Golden et al. 1998). Sea ice is considered permeable for fluid transport when the brine volume exceeds this threshold (Tedesco et al. 2010; references therein). The modified BFM-SI was applied to data collected during a spring ice algal bloom of the Coronation Gulf in the Canadian Arctic (Campbell et al. 2016, 2017; Fig. 1). From this we evaluate the impact of improving the light transmission representation on algal chl *a* accumulation in the bloom (Fig. 6). Output of the thermo-halodynamic component of the BFM-SI model with and without using field observations are shown in Fig. 6a–c. The model is initialised from the start of August when there is no sea ice in the region, and forced with the reanalysis data or field observations. In the *NCEP* + *observations*-simulation, the snow depth is limited to a maximum of 5 cm prior to the start of the observations in order to better relate the observed snow depth of 6 cm measured at the start of the observation period.

### Results from the modified BFM-SI

Nudging the modelled ice thickness and snow depth to the observed values in this case study significantly changes the model output, delaying the ice melt onset by 34 days (Fig. 6a) and snow cover melt by 33 days (Fig. 6b). The result is a more realistic light environment in the BAL of the sea ice (Fig. 6c). The earlier melt onset determined from only the reanalysis data as a forcing is potentially a bias due to the large grid cell size in the data. That is, the sampling location is in a comparatively narrow (30 km wide) strait surrounded by land (Fig. 1), and thus the model defining the atmospheric forcing likely treats the location as a land versus ocean grid cell due to land representing a majority of the spatial grid cell cover (approximately 1.9° in latitude and longitude). From this work we see that it is not a trivial task to accurately represent the physical environment of snow and sea ice that are controlling the biogeochemical model processes. It is also evident here and from previous model studies (e.g. Mortensen et al. 2017) that using field observations, or alternatively, the output from coupled models with high enough spatial resolution (Watanabe et al. 2019), can drastically improve the forcing for process model studies of physical-biogeochemical conditions of sea ice habitats. This is especially true for complex shorelines characteristic of archipelagos like this study region in the lowermost Northwest Passage, which are coincidentally also likely to experience greater anthropogenic disturbance in the future with sea ice retreat and increases in shipping traffic.

Total modeled chl *a* of the ice algal bloom (Fig. 6d) decreased after modeled nitrate concentrations in the BAL become depleted (data not shown), indicating nutrient depletion in the system that is supported by observations of Campbell et al. (2016) in the study region. In contrast, observed chl *a* concentration increased over the study period. One possible reason for this discrepancy is the even distribution of algae over the BAL in the modeled simulations, while in the field, the algal communities are typically concentrated in the bottommost millimeter of the ice as a result of greater access to nutrients from the water column (Campbell et al. 2018). As outlined previously, the vertical position of bottom-ice algae can be highly variable. For example, the location of the coloured band in Fig. 5a shows a different type of algal distribution in sea ice of northwestern Hudson Bay (i.e. it is higher up in the ice) than what was assumed here for the Coronation Gulf. This difference adds complexity to comparison of model results between regions or individual studies. Nevertheless, the modeled accumulation of chl *a* (Fig. 6d) shows that both increasing and decreasing the BAL irradiance shifts the timing of the peak of the algal bloom (i.e. maximum chl *a*), but results in similar peak magnitudes. The model setup used here includes a single group of algae with fixed photophysiological parameterisations. Including additional algal groups that were potentially more efficient in utilising nutrients under varying light levels could result in the modelled chl *a* better following observed concentrations. This is an important next step in development of the BFM-SI.

## Societal and policy implications

Sea ice microorganisms provide valuable ecosystem services to society by supporting a marine food web of consumers that are available for harvest by global fisheries and Arctic Indigenous peoples, as well as aesthetic-based experiences (e.g. ecotourism). Through photosynthetic activity, ice algae also impact the global cycling of carbon and help remove rising anthropogenic CO_2_ levels in the atmosphere (Steiner et al. 2021; Lannuzel et al. 2020). Polar amplification of global warming (Meredith et al. 2019) combined with additional stressors in the Arctic like increased shipping traffic (Aksenov et al. 2017) will increasingly impact the habitat conditions of sea ice microorganisms, and thus the services they provide. It is thus critically important that the *sustainable use and preservation of marine and coastal ecosystems* (UN Sustainable Development Goal 14; Jensen 2020) in northern latitudes include knowledge on sea ice habitats and their microbial residents. However, this is no trivial task provided the large expanse and remoteness of sea ice covered regions, difficulties inherent to work in the challenging conditions they personify, and the rate of environmental change already occurring in the Arctic. It is only through methodological and sensor-based innovation, as well as refining of predictive biogeochemical models, that a sufficient knowledge base to inform on the management of sea ice ecosystems and their associated waters is created. Activities of the DiatomARCTIC project outlined here have contributed to such knowledge; development of molecular techniques to assess true microbial diversity, new application sensor-based measurements of chl *a* biomass variability, use of the BFM-SI model to represent environmental drivers of phenology in ice algal blooms. However, continued investment by governments into technological developments that effectively document and understand microbial abundance, function and activity in sea-ice covered regions of the Arctic is critical to accomplishing this goal in the future. Within the IPBES conceptual framework, development and government support for new technologies is stated as a key anthropogenic asset in addressing climate change through the strengthening of nature's contributions to people and ultimately improving overall quality of life (Diaz et al. 2015).

## Conclusions

Global warming has created a pressing need to effectively characterise the habitats and microbial communities of Arctic marine ecosystems. To best inform on the management of the Arctic marine ecosystem it is critically important that this work includes the unique environment of sea ice, which hosts an active and microbially diverse community that provides a number of services for northern latitudes and the global community. Our ability to prepare for change and manage resources accordingly is hindered by the sparse coverage of sea ice biogeochemical studies across the vast Arctic, and to some extent, the caveats of traditional methodologies that may be inaccurate, time consuming, or limited by gaps in our knowledge of such complex systems. The compiled work of the DiatomARCTIC project, which drew upon data over a broad geographical range in the Arctic, has demonstrated through case studies how innovative approaches of combining in-situ, laboratory and numerical studies may advance our understanding of microbial function and growth within sea ice. The use of molecular tools like metagenomics and metatranscriptomics has the capacity to reveal complex and novel metabolic strategies within sea ice environments. Here we have shown that this includes the potential for facultative anaerobic activity by bacteria, which has important implications for nutrient cycling in the ice and potentially on nutrient availability to algal communities. Through representation of chl *a* using optical or photometric approaches we have demonstrated the potential to assess ice algal chl *a* biomass over scales of significantly greater spatial coverage or detail that are not ordinarily possible with collection of ice cores alone. Finally, our ice algal bloom simulations using the BFM-SI model highlight the critical importance of validating environmental conditions parameterized by models to ensure accurate representation of bloom development for process studies of individual field sites. The complexity and severity of conditions in the Arctic marine system presents an enormous challenge in scientific investigations. In addition, strong regional and seasonal differences will require more advances in up-scaling observations for accurate representation of biogeochemical processes in model settings and basin-wide estimates. Through development of innovations to tackle these challenges we can create an accurate benchmark of understanding, from which the consequences of future change to the system may be evaluated by researchers and governing bodies alike.

## Supplementary Information

Below is the link to the electronic supplementary material.

Supplementary file1 (PDF 318 KB)
