# Supplementary material for: Monitoring a changing Arctic: Recent advancements in the study of sea ice microbial communities
Source: Ambio. 2021 Nov 25;51(2):318–32. doi: 10.1007/s13280-021-01658-z (PMC8692635; doi:10.1007/s13280-021-01658-z)
Supplement: Supplementary file 1 — Supplementary file1 (PDF 318 KB) [file 13280_2021_1658_MOESM1_ESM.pdf]

Supplementary Material

Monitoring a changing Arctic: Recent advancements in the study of sea ice microbial communities

K Campbell[1,2], I Matero[3], C Bellas[2], T Turpin-Jelfs[2], P Anhaus[3], M Graeve[3], F Fripiat[4], M Tranter[2,5], JC Landy[2,6], P Sanchez-Baracaldo[2], E Leu[7], C Katlein[3], CJ Mundy[8], S Rysgaard[6,8,9], L Tedesco[10], C Haas[3], M Nicolaus[3]

[1]Department of Arctic and Marine Biology, UiT The Arctic University of Norway, Tromsø, Norway
[2]Bristol Glaciology Centre, School of Geographical Sciences, University of Bristol, Bristol, UK
[3]Alfred-Wegener-Institute Helmholtz Centre for Polar and Marine Research, Bremerhaven, Germany
[4]Department Geosciences, Environment and Society, Université Libre de Bruxelles
[5]Arctic Research Centre, Department of Bioscience, University of Aarhus, Aarhus, Denmark
[6]Department of Physics and Technology, UiT The Arctic University of Norway, Tromsø, Norway
[7]Akvaplan-niva AS, CIENS, Gaustadalleen 21, 0349 Oslo, Norway
[8]Centre for Earth Observation Science, University of Manitoba, Winnipeg MB, Canada
[9]Greenland Climate Research Centre, Nuuk, Greenland
[10]Finnish Environment Institute, Helsinki, Finland

Supplementary Material

S.1 References of primary and bacterial production

Data from the following references are represented in Figure 1:

General Movement of Surface Waters

Stein, R., and R.W. Macdonald. 2004. The Organic Carbon Cycle in the Arctic Ocean. Springer.

Nutrient Fluxes

Torres-Valdés, S., Tsubouchi, T., Bacon, S., Naveira-Garabato, A. C., Sanders, R., McLaughlin, F. A., Petrie, B., Kattner, G. et al., 2013. Export of nutrients from the Arctic Ocean. *Journal of Geophysical Research: Oceans* 118(4): 1625–1644. https://doi.org/10.1002/jgrc.20063

Primary Production

Booth, J.A. (1984), The epontic algal community of the ice edge zone and its significance to the Davis Strait Ecosystem, Arctic 37(3), 234-243.

Campbell, K., Mundy, C. J., Landy, J. C., Delaforge, A., Michel, C., & Rysgaard, S. (2016). Community dynamics of bottom-ice algae in Dease Strait of the Canadian Arctic. *Progress in Oceanography*, *149*, 27-39. https://doi.org/10.1016/j.pocean.2016.10.005

Cota, G., and R.E.H. Smith (1991), Ecology of bottom ice algae: II, Dynamics, distributions and productivity, *J. Mar. Syst*. 2, 279-295, doi: 10.1016/0924-7963(91)90037-U.

Fernández-Méndez, M., Katlein, C., Rabe, B., Nicolaus, M., Peeken, I., Bakker, K., Flores, H., and Boetius, A.: Photosynthetic production in the central Arctic Ocean during the record sea-ice minimum in 2012, Biogeosciences, 12, 3525–3549, https://doi.org/10.5194/bg-12-3525-2015, 2015.

Gosselin, M., Legendre, L., Demers, S., and R.G. Ingram (1985), Responses of sea-ice microalgae to climatic and fortnightly tidal energy inputs (Manitounuk Sound, Hudson bay), *Can. J. Fish. Aquat. Sci*. 42, 999-1006.

Herman, A.W., Knox, D.F., Conrad, J., and M.R. Mitchell (1993), Instruments for measuring sub-ice algal profiles and productivity in situ, *Can. J. Fish. Aquat. Sci*. 50, 359-369, doi: 10.1139/f93-041.

Johnsen, G., and E.N. Hegseth (1991), Photoadaptation of sea-ice microalgae in the Barents Sea, *Polar Biol.* 11, 179-184, doi: 10.1007/BF00240206.

Kaartokallio, H. (2004), Food web components, and physical and chemical properties of Baltic Sea ice, *Mar. Ecol. Prog. Ser.* 273, 49-63, doi:10.3354/meps273049.

Lee, S.H., de Mora, S.J., Gosselin, M., Levasseur, M., Bouillon, R.-C., Nozais, C., and C. Michel (2001), Particulate dimethylsulfoxide in Arctic sea-ice algal communities: the cryoprotectant hypothesis revisited, *J. Phycol.* 37, 488-499, doi: 10.1046/j.1529-8817.2001.037004488.x.

Mock, T., and R. Gradinger (1999), Determination of Arctic ice algal production with a new in situ incubation technique, *Mar. Ecol. Prog. Ser.* 177, 15-26, doi: 10.3354/meps177015.

Smith, R.E.H., and A.W. Herman (1992), In situ patterns of intracellular photosynthate allocation by sea ice algae in the Canadian High Arctic, *Polar Biol.* 12, 545-551, doi: 10.1007/BF00236978.

Bacterial Production

Bunch, J.N., and R.C. Harland (1990), Bacterial production in the bottom surface of sea ice in the Canadian subarctic, *Can. J. Fish. Aquat. Sci*. 43, 1986-1995, doi: 10.1139/f90-223.

Haecky, P., and A. Anderson (1993), Primary and bacterial production in sea ice in the northern Baltic Sea, *Aquat. Microbiol. Ecol.* 20, 107-118, doi: 10.3354/ame020107.

Kaartokallio, H., Søgaard, D.H., Norman, L., Rysgaard, S., Tison, J.L-., Delille, B., and D.N. Thomas (2013), Short-term variability in bacterial abundance, cell properties, and incorporation of leucine and thymidine in subarctic sea ice, *Aquat. Microbiol. Ecol.* 71, 57-73, doi:10.3354/ame01667.

Maranger, R., Vaqué, D., Nguyen, D., Hébert, M-P., and E. Lara (2016), Pan-Arctic patterns of planktonic heterotrophic microbial abundance and processes: Controlling factors and potential impacts of warming, *Prog. Oceanog*., doi: 10.1016/j.pocean.2015.07.006.

Mock, T., Meiners, K.M., and H.C. Giesenhagen (1997), Bacteria in sea ice and underlying brackish water at 54" 26' 50" N (Baltic Sea, Kiel Bight), *Mar. Ecol. Prog. Ser.* 158, 23-40, doi:10.3354/meps158023.

Smith, R.E.H., and P. Clement (1990), Heterotrophic activity and bacterial productivity in assemblages in microbes from sea ice in the High Arctic, *Polar Biol.* 10, 351-357, doi: 10.1007/BF00237822.

Søgaard DH, Kristensen M, Rysgaard S, Glud RN, Hansen PJ, and K.M Hilligsoe (2010), Autotrophic and heterotrophic activity in Arctic first-year sea ice: seasonal study from Malene Bight, SW Greenland, *Mar. Ecol. Progr. Ser*. 419: 31-45, doi: 10.3354/meps08845.

Søgaard, D.H., Thomas, D.N., Rysgaard, S., Glud, R.N., Norman, L., Kaartokallio, H., Juul-Pedersen-Juul, T., and N.-X Gelfius (2013), The relative contributions of biological and abiotic processes to carbon dynamics in subarctic sea ice, *Polar Biol.* 36(12), 1761-1777, doi: 10.1007/s00300-013-1396-3.

S.2 Northwestern Hudson Bay dataset

Data collected during the The Coral Harbour Oceanographic Observation and Sea ice Experiments (CHOOSE) from May 1 – June 7, 2019, took place in northwestern Hudson Bay, offshore from the community of Coral Harbour. Sampling focused on the bottom 10 cm of ice cores collected from three study sites along a transect orientated perpendicular to the flow edge a polynya and the Canadian coastline. Specific location and dates of sampling are detailed in Table S.1. Three sites (A, C, and F) situated increasingly offshore from the community of Coral Harbour, Nunavut were sampled in 2019 during the spring bloom period on 4 May (Site C), 17 May

(sites A, C & F) and 29 May (Site C). The >10 µm size fraction was chosen to reduce the signal of the algal bloom, allowing for amplicons of larger grazers to be detected.

Table S.1 Location of ice cores drilled for 18S rRNA gene analysis.

Site	Location	Date	Snow depth (cm)	18S rRNA sequences
A	64°06'52.6"N 83°04'45.8"W	17/5/19	15	18-21K
C	63°59'39.1"N 83°20'19.1"W	05/05/19<br>17/5/19<br>30/5/19	9<br>23.5<br>13	14-16K<br>14-20K<br>14-21k
F	63°54'36.7"N 83°18'24.6"W	17/5/19	19	16-20k

18S rRNA gene analysis: From each ice core, the bottom 10cm of core was melted and filtered onto triplicate 47mm, 10 µm filters. DNA was extracted from the filters using a DNeasy Plant Mini Kit (Qiagen). We amplified the V4 region of the 18S rRNA gene as previously described (Stoeck et al., 2010), yielding 500 basepair fragments. PCR products were sequenced on an Illumina MiSeq (Nano V2, 2 ╳ 250bp paired end reads) by the Bristol Genomics Facility. Amplicons were denoised using DADA2 (Callahan et al 2016) in the QIIME2 package (Estaki et al., 2020). Sequences were assigned a taxonomy using the QIIME2 classifier pretrainted on Silva 138 99% OTUs (Bokulich et al., 2018; Quast et al., 2013).

Metagenomic assembled genome analysis: Metagenomes were generated as previously described, from a pooled sample across all sites (Table S.1) (Bellas et al., in prep). Briefly, the bottom 10cm of ice core was melted, prefiltered through a 10 µm filter before being filtered onto a 90mm, 0.2um polycarbonate filter to collect prokaryotes. This filter was frozen and stored at -80°C. DNA was extracted from the filtered with a DNeasy PowerWater Kit (Qiagen) before being sequenced on an Illumina NextSeq 500 at the Bristol Genomics Facility. Reads were assembled using MEGAHIT (Li et al., 2015), binned using METABAT2 (Kang et al., 2019) and taxonomically classified using GTDB-Tk (Chaumeil et al., 2020). Functional annotation of the individual MAGs was carried out by searching against the Kyoto Encyclopedia of Genes and Genomes (KEGG), using the GhostKOALA (Kanehisa et al., 2016) (www.genome.jp/kegg/).

References for S.2

Bokulich, N. A., Kaehler, B. D., Rideout, J. R., Dillon, M., Bolyen, E., Knight, R., Huttley, G. A., & Gregory Caporaso, J. (2018). Optimizing taxonomic classification of marker-gene amplicon sequences with QIIME 2's q2-feature-classifier plugin. Microbiome, 6(1), 90. doi.org/10.1186/s40168-018-0470-z

Chaumeil, P.-A., Mussig, A. J., Hugenholtz, P., & Parks, D. H. (2020). GTDB-Tk: A toolkit to classify genomes with the Genome Taxonomy Database. Bioinformatics, 36(6), 1925–1927. doi.org/10.1093/bioinformatics/btz848

Estaki, M., Jiang, L., Bokulich, N. A., McDonald, D., González, A., Kosciolek, T., Martino, C., Zhu, Q., Birmingham, A., Vázquez-Baeza, Y., Dillon, M. R., Bolyen, E., Caporaso, J. G., & Knight, R. (2020). QIIME 2 Enables Comprehensive End-to-End Analysis of Diverse Microbiome Data and Comparative Studies with Publicly Available Data. Current Protocols in Bioinformatics, 70(1), e100. doi.org/10.1002/cpbi.100

Kanehisa, M., Sato, Y., & Morishima, K. (2016). BlastKOALA and GhostKOALA: KEGG Tools for Functional Characterization of Genome and Metagenome Sequences. Journal of Molecular Biology, 428(4), 726–731. doi.org/10.1016/j.jmb.2015.11.006

Kang, D. D., Li, F., Kirton, E., Thomas, A., Egan, R., An, H., & Wang, Z. (2019). MetaBAT 2: An adaptive binning algorithm for robust and efficient genome reconstruction from metagenome assemblies. PeerJ, 7. doi.org/10.7717/peerj.7359

Li, D., Liu, C.-M., Luo, R., Sadakane, K., & Lam, T.-W. (2015). MEGAHIT: An ultra-fast single-node solution for large and complex metagenomics assembly via succinct de Bruijn graph. Bioinformatics (Oxford, England), 31(10), 1674–1676. doi.org/10.1093/bioinformatics/btv033

Quast, C., Pruesse, E., Yilmaz, P., Gerken, J., Schweer, T., Yarza, P., Peplies, J., & Glöckner, F. O. (2013). The SILVA ribosomal RNA gene database project: Improved data processing and web-based tools. Nucleic Acids Research, 41(D1), D590–D596. doi.org/10.1093/nar/gks1219

Stoeck, T., Bass, D., Nebel, M., Christen, R., Jones, M. D. M., Breiner, H.-W., & Richards, T. A. (2010). Multiple marker parallel tag environmental DNA sequencing reveals a highly complex eukaryotic community in marine anoxic water. Molecular Ecology, 19(s1), 21–31. doi.org/10.1111/j.1365-294X.2009.04480.x

S.3 Lincoln Sea dataset

Data were collected as part of the *Multidisciplinary Arctic Program - Last Ice (MAP- Last ice)* in the Lincoln Sea (82.576°N -62.471°W), located off-shore from the Canadian Forces Station *Alert.* This was done between 7 and 23 May, 2018. Additional information can be found in:

Campbell, K., Lange, B., Landy, J.C., Katlein, C., Nicolaus, M., Anhaus, P., Matero, I., Gradinger, R., Charette, J., Duerksen, S., Tremblay, P., Rysgaard, S., Tranter, M., Haas, C., and C. Michel (*in review*). Widespread net heterotrophy in High Arctic first-year and multi-year sea ice. *Elementa*.

S.4 Lowermost Northwest Passage dataset

Data were collected in Spring 2014 on 12 discrete sampling occasions between 21 April and 9 June from landfast first-year sea ice. The sampling site in Dease Strait (69.017°N 105.317°W) was near Cambridge Bay, Nunavut, Canada. The data used in this study is a subset of the more comprehensive dataset described in Campbell et al. (2016).
